# Supplementary material for: Child sexual abuse and its determinants among children in Addis Ababa Ethiopia: Systematic review and meta-analysis
Source: PLOS Glob Public Health. 2024 Apr 3;4(4):e0001979. doi: 10.1371/journal.pgph.0001979 (PMC10990560; doi:10.1371/journal.pgph.0001979)
Supplement: Attachment — Submitted filename: Point by point response.docx [file pgph.0001979.s002.docx]

**Point by point response for editors/reviewers comments**

**PLOS Global Public Health**

**Manuscript title:** **Child Sexual Abuse and Its determinants Among Children in Ethiopia: Systematic review and Meta-analysis**

**Manuscript ID:** **PGPH-D-23-00743**

**Dear editor/reviewer.**

Dear all,

We would like to thank you for this constructive, building, and improvable comments on this manuscript that would improve the content of the manuscript. We considered each comment and clarification questions of editors and reviewers on the manuscript thoroughly. Our point-by-point responses for each comment and question are described in detail on the following pages. Further, the details of changes were shown by track changes in the supplementary document attached.

**Response to editors’ comment**

We noticed you have some minor occurrence of overlapping text with the following previous publication(s), which needs to be addressed:

**Authors’ response**: Thank you for editor for the comment. We have addressed the comment. (**See the revised manuscript**)

**Response to reviewers’ comment**

**Reviewer#1**

1. The 5th paragraph from the introduction, this sentence is incomplete “Child sexual abuse has long-lasting effects and overwhelming on a child” Overwhelming what?

**Authors’ response**: Thank you for reviewer for the comments. We have edited the sentence and make it meaningful. (**See the revised manuscript**)

2. In the study characteristics, what was the researchers quality assessment based upon?

**Authors’ response:** Thank you reviewer for the comments. We have highlighted the method of quality assessment of the included studies in the data extraction and quality assessment section of the manuscript **(See the Method section, Page 6-7)**

3. The information in the result section are inadequate. I would expect this researcher to provide more detailed information on what was found and some of the reasons to the variations in the prevalence of child sexual abuse across different and even some same location of Ethiopia. More so, I expected that the gravity of the determinants of this act would be discussed in details especially to reveal what the commonest determinants were in that order. same with the results on the perpetrators and consequences. The researcher should provide details on how many studies identified what factors or perpetrators or consequences, this will be very relevant to this study.

**Authors’ response:** Thank you reviewer for the comments. As per your suggestion we have addressed the comment. **(See the revised manuscript)**

**Minor points**

4. I would also suggest the review of the repetition of same words at the beginning of every paragraph. This should be reviewed.

**Authors’ response:** Thank you reviewer for the comments. We had extensively modified the entire manuscript with the help of language experts. **(See the revised manuscript)**

**Reviewer#2**

**Major revisions**

1. There was high statistical heterogeneity (I2= 99.5%) in the meta-analysis, but sources were not explored through subgroup analysis or meta-regression based on study-level factors like location, sample, etc. Examining potential sources will clarify if the heterogeneity impacts the overall pooled estimate.

**Authors’ response:** Thank you for the comments, we have addressed all the comments. **(See the revised manuscript)**

2. The authors identified several determinants from their narrative synthesis, but should consider meta-analyzing these if enough studies examined the same factors consistently. This will provide pooled effect sizes rather than just a descriptive summary.

**Authors’ response:** Thank you reviewer for the comment. We’ve addressed the comment. **(See the revised manuscript)**

3. The limitations around generalizability need to be expanded, given the small number of studies from a limited geographic area. Discussion of regional differences would strengthen this point.

**Authors’ response:** Thank you reviewer for the comments. We have addressed it. **(See the revised manuscript)**

**Minor revisions**

1. Some previous studies require fuller citations in the discussion.

**Authors’ response:** Thank you reviewer for the comments. We’ve addressed the comment. **(See the revised manuscript)**

2. Some minor edits to improve writing clarity and flow would further enhance readability.

**Authors’ response:** Thank you reviewer for the comment. We have addressed all the comments. We had extensively edited the entire manuscript for grammatical errors, sentence structure and typographical errors with the help of language experts at the University (**See the revised manuscript**).

3. Biases inherent in child sexual abuse research should be briefly mentioned regarding how they may impact prevalence estimates.

**Authors’ response:** Thank you reviewer for the comments. We have mentioned it. (See the revised manuscript)
