# Supplementary material for: Child sexual abuse and its determinants among children in Addis Ababa Ethiopia: Systematic review and meta-analysis
Source: PLOS Glob Public Health. 2024 Apr 3;4(4):e0001979. doi: 10.1371/journal.pgph.0001979 (PMC10990560; doi:10.1371/journal.pgph.0001979)
Supplement: Attachment — Submitted filename: Response to reviewers_Letter.docx [file pgph.0001979.s003.docx]

**Author’s Point-by-point response letter to reviewer**

**Child Sexual Abuse and Its determinants Among Children in Ethiopia: Systematic review and Meta-analysis**

Birye Dessalegn Mekonnen and Sintayehu Simie Tsega

Corresponding author:

Sintayehu Simie Tsega

Email:[[simies952@gmail.com](mailto:simies952@gmail.com)](mailto:birye22@gmail.com)

January 13, 2024

**To PLOS Global Public Health**

Dear Sir/Madam,

This is a point-by-point response letter that accompanies the responses for the editors and reviewers’ comments concerning the manuscript entitled “Child Sexual Abuse and Its determinants Among Children in Ethiopia: Systematic review and Meta-analysis”. It is known that the manuscript has been reviewed by reviewers and sent back to us the authors to carry out the corrections to meet the editor and reviewers’ concern, and for resubmission.

As the authors of this manuscript, we highly appreciate the insightful comments and concerns raised by the reviewers. These inputs have significantly contributed to enhancing the quality and scientific plausibility of the manuscript. To do so, we have tried to address all the reviewers’ concerns point by point as described below. Therefore, we are pleased to resubmit the revised version of the manuscript for further process and facilitation of its publication on PLOS Global Public Health.

Thank you so much, all the reviewers and editors for your time and providing us these valuable comments and suggestions.

**Author’s Point-by-point response to Reviewers’ Comments**

**Journal Requirements:**

1. Please review your reference list to ensure that it is complete and correct. If you have cited papers that have been retracted, please include the rationale for doing so in the manuscript text, or remove these references and replace them with relevant current references. Any changes to the reference list should be mentioned in the rebuttal letter that accompanies your revised manuscript. If you need to cite a retracted article, indicate the article’s retracted status in the References list and also include a citation and full reference for the retraction notice.

**Authors’ response:** We checked the references which are appropriately cited, and no retracted references cited. No changes have been made on references.

2. We noticed that you used "unpublished" in the manuscript. We do not allow these references, as the PLOS data access policy requires that all data be either published with the manuscript or made available in a publicly accessible database. Please amend the supplementary material to include the referenced data or remove the references.

**Authors’ response:** We used the word "unpublished" to indicate an article that has not been peer reviewed but a publicly accessible on Google. We change the "unpublished" to “non-peer reviewed but a publicly available” in the manuscript.

3. We have noticed that you have uploaded Supporting Information files, but you have not included a list of legends. Please add a full list of legends for your Supporting Information files after the references list.

**Authors’ response:** Now we included a full list of legends for the Supporting Information files after the references list.

**Additional Editor Comments (if provided):**

There is a need to ensure that the title indicates that the studies reviewed are largely from Addis Ababa and not the whole of Ethiopia.

**Authors’ response:** Dear editor, first of all thank you very much for your interest for reviewing our paper and providing constructive and fruitful comments as well as a second chance to revise our manuscript. Though we tried to update the title to indicates that the studies reviewed are largely from Addis Ababa, we couldn’t come up with well described or satisfactory title. After many trials and discussions, we conclude that we need to indicate that the studies reviewed are largely from Addis Ababa in the manuscript but to keep the title as it is. Thus, we tried to indicate that most studies were conducted in Addis Ababa in the manuscript on page 9, line 181-182 and page 16, line 288. But we are happy to accept your suggestion if you still feel that the title should be modified.

Can you indicate how the response rate was calculated and indicate whether the response rate mentioned above includes quantitative and mixed methods studies?

**Authors’ response**: Dear editor, again thank you very much. We were calculated the response rate by dividing the total number of participants (sum of participants in each study) to the total sample size (sum of estimated sample size in each study) and multiplied by 100, for both quantitative and mixed methods studies. However, now we removed the response rate in the manuscript because calculating cumulative response rate is not much necessary for the meta-analysis and the response rate in mixed method studies may mislead readers.

**Determinants of child sexual abuse**

There is likely an interaction between the variables, alcohol use, khat chewing and gender.  I take that it is the boys who are more likely to engage in these behaviours.  This needs to be discussed.

**Authors’ response**: Again, thank you very much. Yes, boys are more likely to engage in the sexual offense behaviours. However, the primary studies couldn’t identify which gender more likely to engage in the sexual offense behaviours. Though the primary studies did not identify which gender more likely to engage in the sexual offenses, we tried to discuss it based on evidence (please see it on page 15, line 270-272 in the manuscript).

**Reviewers' comments:**

**Reviewer #1:** Review: Child Sexual Abuse and Its determinants Among Children in Ethiopia: Systematic review and Meta-analysis

This systematic review and meta-analysis on child sexual abuse among children in Ethiopia make a significant and commendable contribution to our understanding of a critical issue. By synthesizing existing studies, the research not only sheds light on the prevalence of child sexual abuse but also identifies determinants, offering valuable insights for policymakers and practitioners. This study serves as a crucial step forward in addressing a sensitive topic, paving the way for targeted interventions and fostering a safer environment for children in Ethiopia.
However, there are few clarifications that needs to be done on this paper, which are listed below;

**Authors’ response:** Dear reviewer, first of all thank you very much for your interest for reviewing our paper and providing constructive and fruitful comments as well as feedback.

1. Perpetrators of child sexual abuse
In your report of findings here, I don’t think you meant to mention family members and school teachers, as the main perpetrators by the pooled prevalence were neighbors and boys/girls. By the way what does the authors mean by boys/girls? I think a better way to report this will be to separate the sex in order to reveal what gender perpetrates more.

**Authors’ response:** Dear reviewer, again thank you very much. We are confidently unable to say boys or girls are more perpetrators because the primary studies reported without specifying which gender more common. However, we tried to add some discussion in the manuscript on page 15, line 270-272. In addition, we made little revision on the main perpetrators as per your suggestion.

2. Determinants of child sexual abuse
According to your review, only gender and abuse of substances were reported to be the determinants of child sexual abuse, this seems to be inadequate, peradventure they are the most prevalent determinants, it is essential to mention other determinant according to the reviewed articles. There are a lot of factors that could predispose to child sexual abuse, how about children who do not use the reported substances?

**Authors’ response:** Dear reviewer, again thank you very much. Based on the previous comments, we did a meta-analysis for the determinants of child sexual abuse for those factors having adequate data and reported consistently across the studies. However, we removed and failed to report other determinants which were inconsistently categorized or being reported only in one primary study. As your suggestion, and now we believe that the determinants of child sexual abuse which were inconsistently categorized or being reported only in one primary study should be included. Therefore, we systematically reviewed and included those determinants of child sexual abuse. Please see the revision made in the manuscript page 13, line 227-233 and page 15, line 257-265.

3. Discussion: In the paragraph on the perpetrators, the second sentence; “This is supported by an existing literature which indicated that children were most commonly perpetuated by family members, neighbors, relatives, and other persons known to the victim child”...perpetuated by what? do you mean to mention sexually perpetuated?

**Authors’ response:** Dear reviewer, again thank you very much. Yes, we meant that children were commonly sexually perpetuated by family members, neighbors, relatives, and other persons known to the victim child. Now we made some revision on this sentence to make it clear as per your suggestion.

4. Limitations and Implication of study, should be discussed under a separate sub-heading.

**Authors’ response:** Thank you very much. we discussed the imitations and Implication of study under a separate sub-heading after your suggestions.

5. In the last paragraph before the conclusion, the second sentence “. Perpetrators of child sexual abuse were identified that are usually committing sexual abuse against children” should be rewritten for clear understanding.

**Authors’ response:** Many thanks for pointing out this. We tried to rewrite the above mentioned sentence in the manuscript.

6. The authors did not discuss any implication of this study on the determinants of child sexual abuse. What are the legislation concerning the use of these substances among children in Ethiopia? What can recommendations based on the findings from this study can help to limit the gravity of the substance use and thus child sexual abuse?

**Authors’ response:** Dear reviewer, again thanks a lot. To our search and knowledge, we did not obtain clear information on legislation regarding substance or drug use among children in Ethiopia. We tried to discuss and put our recommendation on this regard in the manuscript, page 17, line 306-309.

7. Other general comments:
Check your page numbering, it wasn’t consistent, also multiple line spacing were used, be consistent on either single-spaced or double-spaced.

**Authors’ response:** Dear reviewer, again thank you very much for your suggestions. We checked and tried to address the page numbering and line spacing. We really would like to thank you for your all valuable and constructive feedback, and devoted time to review our work.

**Reviewer #2: General comments:**

The authors have carefully addressed previous reviewer comments and made substantial improvements to the manuscript overall. The introduction frames the importance of child sexual abuse and rationale for the systematic review and meta-analysis well. The methods and analysis appear rigorous, with clear reporting of the search, inclusion criteria, quality assessment, data analysis, and risk of bias procedures. Limitations regarding generalization have been expanded appropriately. I have only minor suggestions to further strengthen the paper:

**Authors’ response:** Dear reviewer, first of all thank you very much for your interest for reviewing our paper and providing constructive and fruitful comments as well as feedback.

Abstract:
- The abstract clearly summarizes key aspects of the study. Consider adding a line on implications/recommendations from the findings to conclude the abstract.

**Authors’ response:** Dear reviewer, again thank you very much. We included the recommendations in the abstract line 51-54.

Introduction:
- Well written with good flow. No specific suggestions.

**Authors’ response:** Thank you very much.

Methods:
- The methods are reported in adequate detail. No further suggestions.

**Authors’ response:** Dear reviewer, thank you so much.

Results:
- The presentation of results is much improved from the initial submission, especially related to perpetrators and consequences of abuse. Consider adding frequencies of how many studies examined each perpetrator/consequence to contextualize percentages.
- For determinants meta-analysis, state reasons if you were unable to pool any identified factors (e.g, inconsistent reporting across studies).

**Authors’ response:** Dear reviewer, again thank you very much. The number of studies examined each perpetrator or determinant are listed under ‘Studies’ column in each table (Table 3 and 4). In addition, we stated the reasons for which we were unable to pool some identified factors of child sexual abuse in the manuscript page 13, line 227-228.

Discussion:
- The discussion covers the central findings and situates them well compared to past literature. To further highlight implications, consider adding another paragraph on tangible next steps/recommendations stemming from this review.
- References are now appropriately incorporated. Just double check journal name abbreviations match reference guidelines.
Minor edits:
- Carefully proofread for any leftover grammatical issues, especially subject-verb agreement.

**Authors’ response:** Many thanks. We made some revision on the discussion section regarding the implications and put recommendations. We also checked grammatical issues and references.

Overall, this systematic review addresses an important public health issue and provides meaningful insight into the magnitude and factors associated with child sexual abuse in the Ethiopian context. With only minor revisions to augment discussion/conclusions, this paper will represent a strong contribution to the growing literature in this area and have potential to impact policy and practice.

**Authors’ response:** Dear reviewer, again thank you very much for your wish and deeds. We really would like to thank you for your all the constructive feedback, and devoted time to review our work.

We look forward to hearing from you at your earliest convenience.

Sintayehu Simie Tsega

Corresponding author
