# Supplementary material for: Child sexual abuse and its determinants among children in Addis Ababa Ethiopia: Systematic review and meta-analysis
Source: PLOS Glob Public Health. 2024 Apr 3;4(4):e0001979. doi: 10.1371/journal.pgph.0001979 (PMC10990560; doi:10.1371/journal.pgph.0001979)
Supplement: Attachment — Submitted filename: Authors response_Letter.docx [file pgph.0001979.s004.docx]

**Author’s Point-by-point response letter to reviewer**

**Title (before revision): Child Sexual Abuse and Its determinants Among Children in Ethiopia: Systematic review and Meta-analysis**

**Title (after revision): Child sexual abuse and its determinants among children in Addis Ababa Ethiopia: Systematic review and Meta-analysis**

Birye Dessalegn Mekonnen and Sintayehu Simie Tsega

Corresponding author:

Sintayehu Simie Tsega

Email:[[simies952@gmail.com](mailto:simies952@gmail.com)](mailto:birye22@gmail.com)

February, 2024

**To PLOS Global Public Health**

Dear Sir/Madam,

This is a point-by-point response letter that accompanies the responses for the editors and reviewers’ comments concerning the manuscript entitled “Child Sexual Abuse and Its determinants Among Children in Ethiopia: Systematic review and Meta-analysis”, which is now rephrased (changed) to “**Child sexual abuse and its determinants among children in Addis Ababa Ethiopia: Systematic review and Meta-analysis”.** It is known that the manuscript has been reviewed by reviewers and sent back to us the authors to carry out the corrections to meet the editor and reviewers’ concern, and for resubmission.

As the authors of this manuscript, we highly appreciate the insightful comments and concerns raised by the reviewers. These inputs have significantly contributed to enhancing the quality and scientific plausibility of the manuscript. To do so, we have tried to address all the reviewers’ concerns point by point as described below. Therefore, we are pleased to resubmit the revised version of the manuscript for further process and facilitation of its publication on PLOS Global Public Health.

**Author’s Point-by-point response to Reviewers’ Comments**

EDITOR:

The revision has addressed all the key comments.  However, there are some minor comments that should be taken into account.  Since almost all papers are from Addis Ababa and not a good representation of Ethiopia as a whole you should consider changing the title, to something like:  **Child sexual abuse and its determinants among children in Addis Ababa Ethiopia: Systematic review and Meta-analysis**

**Authors’ response:** Dear editor, first of all thank you very much for your interest for reviewing our paper and providing constructive and fruitful comments as well as a repeated chance to revise our manuscript. We rephrased or changed the title as per your suggestion.

**Other revisions are:**

**Line 178: Check sentence construction**

**Authors’ response**: Dear editor, again thank you very much. We made some modification on the sentence (page 8, line 170-172).

**Lines 229 to 231:** There is likely an interaction between the variables, alcohol use, khat chewing and gender.  I consider that it is the boys who are more likely to engage in these behaviours.  This needs to be discussed.

**Authors’ response**: Dear editor, again thank you. We discussed that the possibility of an interaction between the variables and the likely of boys to engage in such behaviours in the results and discussion section of the manuscript (page 13, lines 212-214 and page 15, lines 245-248).

I agree with the decision to leave out the response rate

**Authors’ response**: Dear editor, again thank you so much. We really would like to thank you for your all the constructive feedback, and devoted time to handle our work.

We look forward to hearing from you at your earliest convenience.

Sintayehu Simie Tsega

Corresponding author
